# MsTargetPeaker: A Quality-Aware Deep Reinforcement Learning Approach for Peak Identification in Targeted Proteomics

**DOI:** 10.1016/j.mcpro.2026.101523

**Published:** 2026-02-02

**Authors:** Chi Yang, Yung-Chin Hsiao, Chi-Ching Lee, Lichieh Julie Chu, Ta-Sen Yeh, Ping-Chang Cheng, Petrus Tang, Jau-Song Yu

**Affiliations:** 1 Molecular Medicine Research Center, Chang Gung University, Taoyuan, Taiwan; 2 Graduate Institute of Biomedical Sciences, College of Medicine, Chang Gung University, Taoyuan, Taiwan; 3 Department of Surgery, Chang Gung Memorial Hospital, Linkou, Taiwan; 4 Department of Computer Science and Information Engineering, Chang Gung University, Taoyuan, Taiwan; 5 Genomic Medicine Core Laboratory, Chang Gung Memorial Hospital, Linkou, Taiwan; 6 College of Medicine, Chang Gung University, Taoyuan, Taiwan; 7 Department of Parasitology, College of Medicine, Chang Gung University, Taoyuan, Taiwan; 8 Molecular Infectious Disease Research Center, Chang Gung Memorial Hospital, Linkou, Taiwan; 9 Department of Otolaryngology-Head and Neck Surgery, Chang Gung Memorial Hospital, Taoyuan, Taiwan; 10 Research Center for Food and Cosmetic Safety, College of Human Ecology, Chang Gung University of Science and Technology, Taoyuan, Taiwan

**Keywords:** targeted mass spectrometry, multiple reaction monitoring, parallel reaction monitoring, deep reinforcement learning, Monte Carlo tree search, peak identification

## Abstract

Targeted mass spectrometry enables precise peptide quantification by identifying high-quality chromatographic peaks for area integration. Automated peak identification remains challenging, particularly for low-abundance targets, because of interference and noise. Existing approaches typically rely on two supervised learning models, one for selecting peak regions and the other for performing downstream quality control in a separate postprocessing step. However, deferring quality assessment to a separate stage may limit the ability to refine peak boundaries in pursuit of improved quality, as the initial selection is performed without explicit awareness of quality-related criteria. In this study, we present MsTargetPeaker, a quality-aware search procedure for identifying peak regions in targeted proteomics data. The method employs a reinforcement learning agent to guide Monte Carlo tree search to efficiently explore chromatograms and localize target peaks while minimizing interference. Peak quality is dynamically assessed during the search *via* a custom-designed reward function, which prioritizes regions with desirable peak characteristics and enables accurate and robust boundary determination. The reward function further incorporates cross-sample consensus profiles of candidate boundaries to improve the identification of low-quality or ambiguous signals. These innovations support fine-grained peak identification, enhancing both peak quality and quantification precision. In addition, the transparent reward calculation allows MsTargetPeaker to generate interpretable diagnostic quality reports, providing comprehensive metrics across transitions, peak groups, and sample replicates. This facilitates efficient detection of problematic cases for manual curation. Collectively, MsTargetPeaker offers a practical advancement toward robust and automated peak identification in targeted proteomics.

## Highlights

• A reinforcement learning agent is trained for multiple reaction monitoring/parallel reaction mnitoring peak boundary picking.
• Peak quality is assessed dynamically and optimized by Monte Carlo tree search.
• Cross-sample consensus profiles guide consistent boundary selection across runs.
• MsTargetPeaker is accurate across dilution ratios and test datasets.
• Interpretable diagnostic reports enable efficient quality control of peak groups.

## Graphical Abstract

**Figure:**
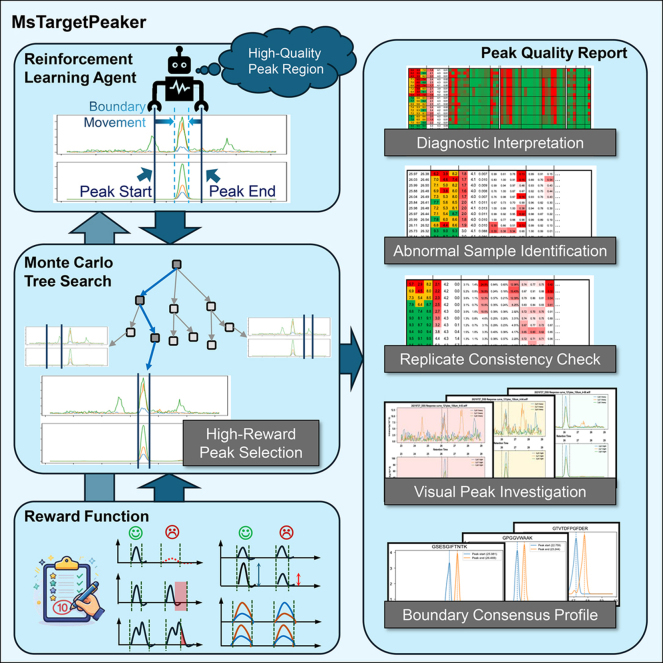

## In Brief

Accurate chromatographic peak boundaries are essential for targeted proteomics but remain difficult to automate for low-abundance or ambiguous signals. MsTargetPeaker performs quality-aware peak identification by combining a deep reinforcement learning agent with Monte Carlo tree search. Peak quality is evaluated during inference using a custom seven-component reward function. Across a response-curve dataset and nine external datasets, MsTargetPeaker increased agreement with manual reference boundaries and improved peak area ratio correlations with manual reference peaks. Transparent scoring also yields diagnostic reports for efficient review and quality control.

Targeted mass spectrometry techniques, such as multiple reaction monitoring (MRM) and parallel reaction monitoring (PRM), can be used to quantify multiple peptides precisely in a single run and have been applied to discover and verify biomarker panels in various clinical studies (1, 2, 3, 4). MRM utilizes instruments such as triple quadrupole mass spectrometers to selectively isolate precursor ions based on their *m/z* ratios, fragment them, and subsequently select the *m/z* ratios of the resulting product ions. In contrast, PRM employs high-resolution instruments such as Q-Orbitrap, where all fragment ions of a selected precursor are simultaneously detected without the need for selective isolation of product ions. By using stable isotope–labeled peptides with known concentrations as the internal standards, the labeled targets (heavy peptides) coelute with endogenous peptides (light peptides) in samples. Peptides can then be quantified by comparing the peak areas of corresponding light and heavy ions. The use of pairs of light and heavy ions, together with sequential *m/z* ratio selection of precursor and fragmented product ions, reduces false positives and enhances the specificity of peptide quantification.

A critical step in precise quantification in targeted mass spectrometry is identifying coeluting signal regions of both light and heavy peptides while effectively excluding interference signals and background noise. Since the selected peak regions serve as the foundation for subsequent peak integration and peptide quantification, incorrectly identified peaks directly lead to inaccurate quantification. Consequently, MRM or PRM data analysis often involves manual evaluation of peak boundaries to eliminate interference and refine target signals for peak area integration. This process is both time consuming and labor intensive, particularly for datasets comprising thousands of chromatograms from MRM assays with hundreds of peptides.

Currently, distinct peaks can often be identified in Skyline (5) using the mProphet scoring model (6), which prioritizes peak groups with consistent coelution patterns and reduced interference. In addition, artificial intelligence (AI)–based methods, including automRm (7) and DeepMRM (8), have been developed to automate and accelerate chromatographic peak picking in targeted mass spectrometry. automRm employs two machine learning models for peak picking and the reporting of qualified peaks for quantification, whereas DeepMRM trains a RetinaNet-based peak detection model that reformulates peak identification as an object detection task (9), along with a convolutional neural network model to classify transition ions unaffected by interference. While these two-stage approaches have advanced the automation of peak boundary selection, the initial selection of peak regions is driven by a supervised model trained primarily to mimic expert annotations, without explicit consideration of peak quality. Although the subsequent model can assess the quality of selected peak regions, rejected peaks may still require manual adjustment to identify more appropriate boundaries. Therefore, there is a need for a more integrated approach that can incorporate peak quality awareness during peak boundary selection, thereby reducing reliance on post hoc adjustment.

In this study, we present MsTargetPeaker, a quality-aware peak search procedure that integrates a deep reinforcement learning agent with Monte Carlo tree search (MCTS) (10, 11). We frame peak picking as a sequential decision problem. The agent is a deep neural network model that reads a snapshot of the chromatograms and outputs two numbers that adjust the peak start and end boundaries. We built a custom Gymnasium environment for agent training (12). Gymnasium provides a standardized environment specification for reinforcement learning and allows us to define how the agent observes chromatograms, adjusts peak boundaries, and receives reward scores. We trained the agent to move the boundaries toward peak regions with higher peak-quality rewards. During inference, MCTS adds a look-ahead mechanism that applies the agent to simulate a few steps of boundary changes and selects a promising path with the highest expected reward. Thus, our proposed approach can dynamically assess peak quality and search for high-quality regions.

A key design is the reward function that guides high-quality peak identification during training and search. We build this function on our previously developed quantitative metrics for peak quality assessment, targeted mass spectrometry quality encoder (TMSQE) scoring (13), and extend it with additional components that capture desirable peak characteristics. TMSQE scoring was established based on a large and diverse dataset covering 22 distinct mass spectrometer models from four major brands (AB Sciex, Agilent Technologies, Thermo Fisher Scientific, and Waters Corporation). We used an unsupervised approach to summarize multiple peak features in this dataset. Higher TMSQE scores indicate that a peak group’s features are closer to the theoretically optimal values. This reward function guides agent training and directs the MCTS search toward high-scoring peak boundaries, especially for low-abundance targets. We evaluated MsTargetPeaker using a response curve dataset spanning a broad concentration range and then assessed its performance on nine publicly available datasets to demonstrate robustness and generalizability across diverse biological conditions. Finally, MsTargetPeaker produces interpretable diagnostic reports through its transparent reward function. These reports help users efficiently identify problematic cases and support quality control in targeted proteomics.

## Experimental Procedures

### Experimental Design and Statistical Rationale

**Fig. 1:**
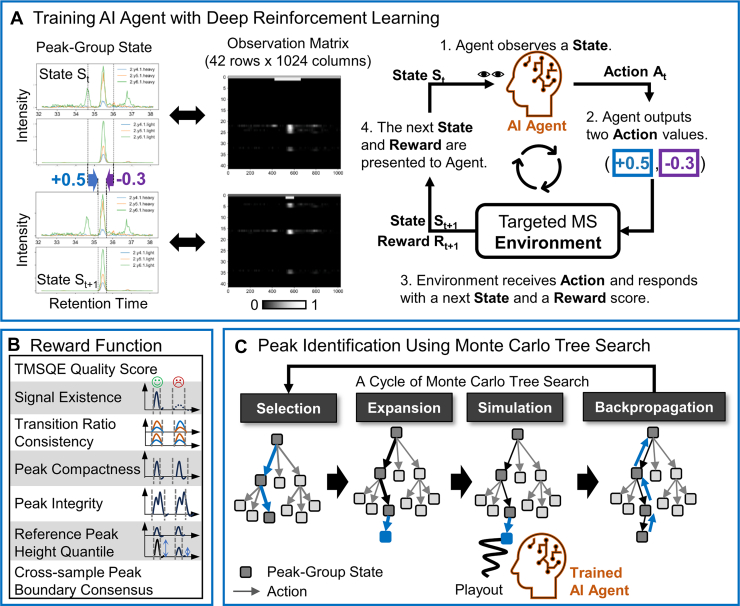
**Design of the quality-aware peak search procedure.***A*, training setup of the reinforcement learning agent to identify optimal peak regions from chromatogram data. The agent observes an observation matrix representing the peak-group state and responds with two action values to control the peak boundary movement, one for each boundary side. After taking the action, the environment responds with the updated matrix and a corresponding reward score, forming a timestep cycle. The reinforcement learning optimizes the agent’s policy, moving peak boundaries to form high-scoring peak regions. *B*, the seven components in the reward function were designed to capture desirable characteristics of target peaks. For five components, the peak illustrations on the *right* depict favored and disfavored characteristics. Because component raw values have different scales and directions, we transform them into directed component scores before combining them. The final score is computed as a multiplicative product of these component scores (Supplemental Fig. S2). Higher final scores indicate more favorable peak regions. *C*, integration of Monte Carlo tree search (MCTS) with double progressive widening (DPW) for dynamic and quality-aware peak identification. MCTS provides a look-ahead mechanism by building a search tree. In this search tree, each node represents a peak-group state, and each edge represents a boundary move action. Starting from an initial peak-group state as the tree root, MCTS grows the search tree by repeating four steps: selection, expansion, simulation, and backpropagation. The algorithm explores candidate boundary moves and evaluates the reward potential of the resulting peak-group states. This evaluation uses a short rollout in which our trained agent adjusts the peak boundaries for up to five steps. Because the set of possible boundary moves from a given state is effectively infinite, DPW limits how many distinct actions are evaluated at each node and allows more actions only as the node is visited more often. After a specified number of cycles, the MCTS output is the highest-scoring peak-group state encountered during the search.

**Fig. 2:**
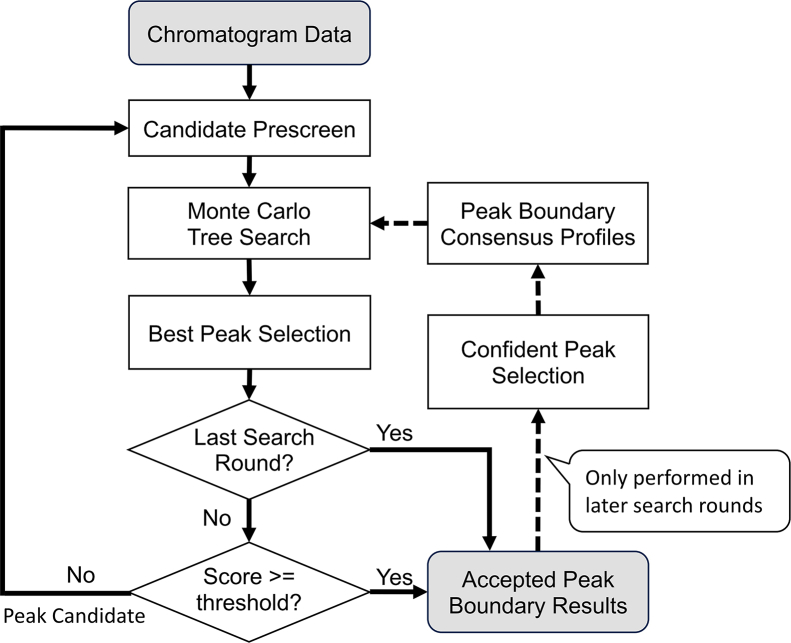
**Peak identification workflow of MsTargetPeaker.** The workflow begins with a prescreen step that generates multiple random peak-boundary candidates and selects the highest-scoring one as the initial peak-group state for Monte Carlo tree search (MCTS). The number of MCTS cycles increases across rounds to enable finer-grained refinement and is a key parameter listed in Table 1. In each round, the best peak candidate identified by the search is accepted if its reward score exceeds the round-specific threshold. Otherwise, the best candidate is carried forward to the next round. In the next round, the prescreen step evaluates the carried candidate together with newly generated random candidates using that round’s reward function. The highest-scoring candidate is selected as the initial peak-group state for the next-round MCTS. After early rounds, confident peaks (accepted peaks that pass the consensus threshold) are used to build a boundary-consensus profile for each target peptide. This consensus profile is incorporated in later rounds to assist peak boundary selection.

**Fig. 3:**
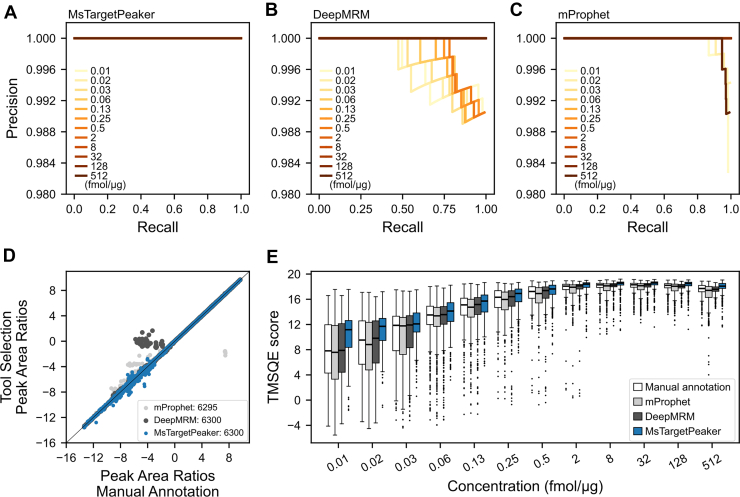
**Comparisons of peak identification on the reverse response curve dataset.** The precision–recall curves for MsTargetPeaker, DeepMRM, and mProphet across a range of concentration levels are presented in *A* to *C*, respectively. *D*, displays the log_2_-transformed peak area ratios between manually annotated peaks (*x*-axis) and accepted tool-selected peaks (*y*-axis), illustrating the concordance between automated peak selection and manual annotation. Next to each tool name, we report the number of accepted peak groups that passed the score cutoff that maximized the F1 score. Last, *E* illustrates the TMSQE quality scores of the resulting peak regions determined by manual annotation, mProphet, DeepMRM, and MsTargetPeaker. The distributions of these scores are depicted in boxplots, where the *boxes* indicate the interquartile range (IQR), and median values are marked by *horizontal lines* within the boxes. The *whiskers* extend to 1.5 times the IQR, and outliers beyond this range are shown as individual dots.

Our goal is to construct a quality-aware search procedure for identifying target peak regions. The procedure consists of three main parts: (1) training a reinforcement learning agent (Fig. 1*A*), (2) designing a reward function to evaluate desirable peak characteristics (Fig. 1*B*), and (3) applying MCTS guided by the trained agent for peak identification (Fig. 1*C*). The peak identification workflow of MsTargetPeaker consists of seven rounds of MCTS and is shown in Figure 2.

The reinforcement learning agent is trained to optimize a boundary adjustment policy and to locate potential peak regions that yield the highest reward scores in chromatograms (Fig. 1*A*). After training, the agent infers boundary movements directly from peak-group states without considering reward scores, as its policy is fixed and no longer responsive to rewards during inference. Here, a peak group is defined as the set of chromatograms for one target in one sample, and a peak-group state corresponds to one peak group. To enable dynamic quality awareness during peak identification, we integrated MCTS and used the trained agent to guide tree expansion toward its preferred decisions. This strategy enables dynamic evaluation of each candidate region during the tree search and aims to select the one with the highest reward score. In this study, MsTargetPeaker performs up to seven rounds of MCTS to progressively identify peaks from peak groups across a range of signal qualities, from clear to ambiguous. The reward function used for both agent training and MCTS includes the first six components shown in Figure 1*B*. As for the last component, the cross-sample boundary consensus is incorporated only during the later MCTS rounds (Fig. 2). This is because the agent is trained on shuffled peak groups without cross-sample information, and this component requires confident peak regions to be identified in earlier rounds to ensure that boundary consensus profiles can be reliably established.

To evaluate the performance of peak identification, we used four metrics: average precision (AP), Pearson’s correlation coefficient (PCC), Spearman’s rank correlation coefficient (SPC), and mean arctangent absolute percentage error (MAAPE). We compared the peak results of MsTargetPeaker with those of mProphet and DeepMRM.

### Collected Datasets for Training and Evaluation

We applied the same targeted proteomics dataset that was previously employed to establish TMSQE scoring functions (13). The dataset comprises 1,703,827 peak groups derived from 4045 unique peptides across 75 studies, and they are publicly available on Panorama Public (14). For training the reinforcement learning agent, we used chromatogram data without predefined peak boundary information. In other words, the agent was not provided with existing peak boundary annotations but instead learned to identify optimal peak regions starting from randomly initialized boundaries during reinforcement learning.

To evaluate peak identification performance, we used a reverse response curve dataset comprising 6300 peak groups, including five replicates of 105 target peptides measured at 12 concentration points ranging from 0.01 to 512 fmol/μg. This dataset was originally introduced in our previous study on TMSQE scoring functions, where it demonstrated the capability of the scoring metrics to reflect peak quality across a broad dynamic range. In the present study, we repurposed this dataset as an independent test set that was not included in the training data to compare the peak-picking performance of MsTargetPeaker and DeepMRM across a range of target concentrations. In addition to the response curve dataset, nine datasets from six independent studies were obtained from Panorama Public to evaluate performance on external data (Supplemental Table S1). The testing datasets were deposited after 2023, whereas the training data were collected before October 2021. The training and testing datasets were obtained from different studies and covered diverse subjects and experimental conditions. Although the peptides partially overlap between the training and testing datasets, the testing data were generated in independent studies and cohorts, supporting an unbiased assessment of generalizability. We compared the peaks identified by mProphet, DeepMRM, and MsTargetPeaker with the reference peaks provided in these datasets.

### Construction of the Reinforcement Learning Environment for Locating Chromatographic Peak Regions

Following the specifications of Gymnasium (12) for reinforcement learning, we implemented a virtual environment that interacts with the agent for peak boundary optimization. At each timestep, the environment (i) constructs an observation matrix representing the current peak-group state, (ii) applies the agent’s action values to adjust the peak boundaries, and (iii) returns a reward score for the updated state.

As shown in Figure 1*A*, each peak-group state is encoded as a 42 × 1024 observation matrix that is provided to the agent at each timestep. The matrix contains peak boundary information (peak start and peak end) and intensity traces from paired light and heavy transitions. Along the row dimension, the 42 rows encode boundary features and transition traces, whereas the 1024 columns span the retention-time axis (see Supplemental Fig. S1 for a detailed illustration). For each peak-group state, the environment constructs this observation matrix and passes it to the agent.

To adjust the retention time boundaries, the environment receives two action values in the range −1 to +1. These two values update the peak start and end boundaries, respectively. Positive values move a boundary to the right along the retention time axis, whereas negative values move it to the left. We set the largest possible boundary shift to 200 retention time points when the absolute action value is 1.0. Smaller action values produce proportionally smaller shifts. For example, an action value of 0.321 shifts the boundary by 64 time points (rounded to an integer). After each update, the environment applies the new boundaries and reconstructs the observation matrix (Supplemental Fig. S1). It then returns a reward score for the updated peak-group state, computed using the reward function.

### Reward Function Design

To guide our AI agent using reinforcement learning as shown in Figure 1*B*, we established a reward function to capture desirable peak characteristics. The reward function contains seven components, including (1) a peak quality score; (2) a binary indicator to flag the existence of potential signals within the selected peak region (SignalExistence); (3) a quantitative measure of the consistency of peak area ratios across transition pairs in the chromatogram (PairRatioConsistency); (4) a quantitative measure of peak integrity (PeakIntegrity); (5) PeakCompactness, quantified by a peak-to-bounding-box area ratio; (6) the quantile of reference peak height within the chromatogram (ReferencePeakHeightQuantile); and (7) a cross-sample boundary consensus score that reflects agreement of boundary positions across samples of the same target peptide.

The first component quantifies peak quality using TMSQE, a set of scoring functions we previously developed (13), based on quality metrics originally introduced in TargetedMSQC (15). TMSQE employs unsupervised variational autoencoders to learn latent representations of peak quality by summarizing 47 derived features spanning nine aspects: jaggedness, symmetry, similarity, modality, shift, full width at half-maximum, area ratio, intensity, and retention time. These scoring functions provide a data-driven and systematic evaluation across three quality types: individual transition quality, overall peak group quality, and transition consistency. In this study, we primarily used the first two to define overall peak quality in individual chromatograms. Since each peak group yields one type I score per transition, we calculated the median score across transitions, considering only the top five transitions when more than five were available. This strategy prevents the agent from selecting peak regions based solely on a single top-scoring transition, thereby enhancing robustness against interference. TMSQE scores serve as the core quantitative metric for evaluating the quality of selected peak regions.

In addition to the peak quality score, the remaining six components capture desirable peak characteristics by addressing signal presence, transition pair ratio consistency, peak integrity, peak compactness, the reference peak height quantile, and boundary consensus across samples. The second component, SignalExistence, compares the local mean intensity within the selected peak region with the average of the three highest chromatogram-wide mean intensities among transition ion traces to identify potential signal regions. SignalExistence equals 1 when the local mean exceeds this chromatogram-wide average and 0 otherwise. The third component, PairRatioConsistency, is a type III TMSQE quality feature that evaluates the consistency of light-to-heavy area ratios across transition ion pairs in the chromatogram. This feature helps exclude coeluted interference signals that exhibit incorrect ordering of area ratios. The fourth component reflects peak integrity through an emphasis on peak shape, as illustrated in Supplemental Fig. S2. For PeakCompactness, the fifth component is defined as the ratio of the peak area to the area of the bounding rectangle enclosing the peak. It constrains the width of the selected peak region, with typical values ranging from approximately 0.3 for broad peaks to 0.6 for narrow peaks. Next, the sixth component is the reference peak height quantile (ReferencePeakHeightQuantile) within the chromatogram for each reference transition ion. The component value is defined as the mean of these quantiles across reference transition ions. Since strong peak signals from reference transition ions are likely derived from target peptides, this component aids in identifying relevant regions, particularly when endogenous light signals are weak or absent because of low analyte abundance. Last, the seventh component, cross-sample boundary consensus, encourages consistent boundary selection across samples. For each target peptide, the probability density functions of peak start and end retention times are estimated using kernel density estimation and normalized to values ranging from zero to one. The consensus score is computed as the average of the normalized densities at the candidate’s start and end positions, rewarding boundary selections that align with commonly observed locations among qualified peaks for the same target. Notably, this component is only incorporated into the reward function during later MCTS rounds.

As these components differ in both scale and the direction associated with better peak characteristics, we applied transformation functions to map each component to a score with a consistent direction (higher is better) (Supplemental Fig. S3). We introduced adjustable exponents on the TMSQE quality score and reference peak height quantile to control the emphasis during the peak search (Table 1). As a result, we first computed a raw reward score as the product of the transformed component scores. We then obtained the final reward score by multiplying the raw reward score by 10 and applying a power transform (exponent = 1.5), as shown in Supplemental Fig. S3. The final reward score (FinalReward) is therefore computed as:

$$\text{FinalReward}={\left({10\cdot \text{TMSQEScore}}^{\text{QualityPower}}\cdot \text{SignalExistence}\cdot \text{PairRatioConsistency}\cdot \text{PeakIntegrity}\cdot \text{PeakCompactness}\cdot {\text{ReferencePeakHeightQuantile}}^{\text{QuantilePower}}\cdot \text{BoundaryConsensus}\right)}^{1.5}$$

**Table 1:** Key search parameters in the seven-round MCTS schedule

Phase	Round	MCTS cycles	Thresholds for downstream search	Noises added to agent's decisions	Use of confident peak boundary consensus	Consensus thresholds to define confident peaks to form consensus	Exponent for TMSQE	Exponent for reference peak height quantile
Search phase	1	300	N/A	10%	No	N/A	2	1
	2	500	8	30%	No	N/A	2	1
	3	1000	6	50%	No	N/A	2	1
	4	1000	4	100%	Yes	4	2	1
Rescue phase	5	1000	2	N/A	Yes	2	2	6
	6	1000	1	N/A	Yes	0.1	1	8
	7	1000	0.01	N/A	No	0.1	1	8

MCTS, Monte Carlo tree search; N/A, not available.

### Reinforcement Learning With Proximal Policy Optimization

Using our prepared training data, the environment initialized each peak-group state by randomly selecting two retention-time points as the initial peak start and peak end boundaries and then constructing the observation matrix for the agent. As shown in Figure 1*A*, the AI agent is a deep neural network that takes the observation matrix as input and outputs two values that represent how to move the peak start and peak end boundaries. The detailed model architecture is provided in Supplemental Table S2. We trained the network using proximal policy optimization (16) implemented in stable-baselines3 (17), so that the agent learned to move the boundaries toward peak regions with higher reward scores. One boundary-update decision corresponds to one timestep (shown as the cycle in Fig. 1*A*), and the agent is allowed up to five adjustment timesteps for each peak-group state. During training, the agent repeatedly observed the matrix, proposed boundary updates, and received reward feedback. These trial-and-error results were used to update the policy so that the agent produced better boundary movements over time. We gradually reduced the learning rate from 1 × 10^−4^ to 1 × 10^−6^, and performance was stabilized after 200 million training timesteps. Training was performed on a Dell Tower 7920 workstation with an NVIDIA RTX A6000 GPU. We used 36 parallel environments to generate training experience more efficiently, achieving an average speed of ∼5 million timesteps per day.

### Peak Identification With MCTS and Double Progressive Widening

To support quality-aware peak identification, we integrated our trained reinforcement learning agent with MCTS and double progressive widening (DPW) (18, 19). MCTS is a planning algorithm that searches for high-reward decisions by constructing a search tree to simulate and explore candidate boundary adjustments toward high-reward peak regions, whereas DPW is an extension that enables MCTS to operate efficiently with continuous boundary-adjustment actions by limiting how many distinct actions are explored at each peak-group state. This MCTS–DPW integration provides an efficient tree-search framework for continuous boundary adjustments in chromatograms.

As shown in Figure 1*C*, we represent the search as a tree. Each node corresponds to a peak-group state with a specific pair of peak boundaries. Each edge corresponds to applying one boundary-adjustment action that generates a new candidate state. Starting from an initial root state, MCTS iteratively grows and refines the tree through the four steps: selection, expansion, simulation, and backpropagation. In each cycle, MCTS selects a promising candidate state, tries a few boundary adjustments to estimate its reward, and then propagates the result back to update the tree.

During selection, the algorithm traverses the tree and selects a node by favoring branches that have produced higher rewards (prefer options that have scored well so far), while still visiting less-explored branches to explore potential boundary adjustments for higher rewards (also try options explored less often). Because there are infinite possible boundary moves for each node, DPW limits how many distinct move options can be added at each node early on. As the node is visited more often, DPW gradually allows more branches. Next, the expansion step adds a new child node to the selected node by applying a newly proposed boundary-adjustment action to that node. The adjustment action can be either proposed by the trained agent or sampled randomly. When using the agent to generate the action, small perturbations as exploration noise can be added to the action values to encourage local exploration of the agent’s decisions.

The simulation step is then used to assess the potential of this newly expanded node by a short playout. Starting from this node, the agent adjusts the boundaries five times at most. Alternatively, a random action sequence can be used to probe regions that may be overlooked by the agent. The accumulated reward score from these simulated adjustments is used to determine the value of this expanded node with high reward potential. The last backpropagation step updates the visit counts and the cumulated rewards by propagating back along the selected node-action path to the tree root node. In the next selection step, DPW uses these visit counts and cumulated rewards to balance the exploration and exploitation, respectively. After multiple cycles, the peak region with the highest reward score within the search history is selected as the final result of the search round.

### Configuration of mProphet and DeepMRM

We compared the peak identification performance of MsTargetPeaker with mProphet and DeepMRM. Unlike DeepMRM and MsTargetPeaker, mProphet requires negative examples for model training, which are often provided by decoy transitions. Because our datasets do not include decoy transitions, we used the built-in reintegration function in Skyline to treat the second-best peaks as negative examples and trained mProphet scoring models to distinguish the best peaks from the second-best peaks. We trained one mProphet model for each dataset. For DeepMRM, we applied the DeepMRM plugin in Skyline to each dataset to perform peak identification without model retraining. Because the DeepMRM plugin does not provide an option to specify the internal standard type (light or heavy transitions as the internal standard) for our reverse response curve dataset, we swapped the light and heavy labels in the transition list file and then used the DeepMRM command-line tool.

### Performance Assessment

We assessed peak-picking performance using three metrics: (1) AP values corresponding to the area under the precision–recall curve, (2) consistency of peak area ratios between tool-identified peaks and reference annotations, and (3) TMSQE quality scores.

We evaluated how well the tool’s selected peak regions matched the reference peak regions using AP. We first computed the intersection-over-union (IoU) between the selected region and the reference region. IoU was defined as the overlap length along the retention-time axis divided by the union length of the two regions. For example, if the two peak regions overlap by 30 s and their union spans 100 s, the IoU is 0.3. A peak call was considered correct when IoU >0.3, following the DeepMRM study.

We then used the scores reported by each tool as confidence scores and applied a series of score cutoffs. For each cutoff, we retained peak calls with confidence scores above the cutoff. Precision was computed as the fraction of retained peak calls that were correct (IoU >0.3), and recall was computed as the fraction of total reference peaks that had a correct retained peak call. Varying the cutoff produced a set of precision–recall pairs that formed the precision–recall curve. AP was computed as the area under the precision–recall curve by trapezoidal integration. Higher AP indicates better overall performance in detecting true target peaks.

Next, we evaluated the concordance between peak area ratios from tool-identified peaks and those from reference peaks using PCC, SPC, and the MAAPE. For each dataset, we selected accepted peaks using the score cutoff that maximized the F1 score based on precision–recall analysis. For each target peptide, peak area ratios were calculated from a single transition as the representative ion, defined as the consistently most intense transition within the reference peak region across samples. We then compared log2-transformed peak area ratios from the accepted tool-identified peaks against those from the reference peaks. Strong correlations and low errors indicate accurate and reliable peak quantification and support the potential to reduce manual effort.

Finally, we assessed peak quality using TMSQE quality scores. To provide an overall quality metric for each peak group, the TMSQE score is calculated as the sum of the median of type I scores and the type II score, representing the median quality of individual transition ions and the overall peak group quality, respectively. As the score of each type ranges from −10 to 10, the summed TMSQE scores ranged from −20 to 20, offering an objective and quantitative measure for peak quality.

## Results

### A Quality-Aware Search Procedure for Automating Target Peak Identification

In this study, we propose MsTargetPeaker to automate the peak identification process using deep reinforcement learning together with MCTS (Fig. 1). The method performs up to seven sequential rounds of MCTS to detect peak regions with varying quality levels. Each round progressively refines the search results, starting from easily distinguishable peaks and proceeding to those affected by strong interference. The first four rounds constitute the search phase, focusing on canonical peaks that exhibit clear coeluted signals from both light and heavy ions. During this phase, the trained agent guides the tree search toward high-scoring candidates. The subsequent rescue phase includes three additional rounds that re-evaluate low-scoring regions. In this phase, since high-scoring canonical peaks were not identified in earlier rounds, the search proceeds without agent guidance and uses random actions to explore alternative boundaries. The overall workflow of MsTargetPeaker is shown in Figure 2.

Multiple search parameters control the search behavior across the seven MCTS rounds to progressively identify peak regions of varying quality. As shown in Table 1, we highlight five key parameters: (1) score thresholds, which determine whether chromatograms should be re-evaluated in the next round; (2) the number of MCTS cycles, which controls the granularity of boundary delineation; (3) exploration noise, a small random perturbation added to the agent’s action values so that it occasionally tries alternative peak boundaries during the MCTS expansion step; (4) component weights in the reward function, which are adjusted across rounds to relax search criteria and accommodate imperfect peaks; and (5) cross-sample boundary consensus, incorporated as the seventh component of the reward function to mitigate the risk of false positives introduced by the relaxed criteria.

The search procedure is re-executed in rounds where peak regions fall below the score threshold, and updated boundaries are retained only if they yield improved scores (Fig. 2). The number of MCTS cycles increases in later rounds to support more fine-grained refinement of peak boundaries, and the exploration noise also increases across rounds to promote broader exploration of nearby boundary candidates. As reward scores are dynamically assessed during the search, the reward function adapts to different signal conditions by adjusting the weights of its components. For example, these adjustments reduce the emphasis on light-to-heavy area ratio consistency and increase the weight on the signal intensities of reference heavy ions, allowing the search to tolerate imperfect peaks. Last, the boundary consensus, derived from qualified peaks with reward scores above a set threshold, guides the search toward regions supported by the majority of cross-sample boundary candidates. Overall, this progressive strategy enables MsTargetPeaker to adaptively identify peak boundaries under varying signal quality and chromatographic complexity.

### Performance of MsTargetPeaker Across a Wide Range of Target Concentration

We evaluated the peak identification performance of MsTargetPeaker using the reverse response curve dataset spanning concentrations from 0.01 to 512 fmol/μg. As shown in Figure 3*A*, MsTargetPeaker achieved an AP value of 1.0 throughout the entire range. In comparison, DeepMRM maintained an AP value of 1.0 only at concentrations above 0.5 fmol/μg and showed reduced performance at lower levels (Fig. 3*B*), and mProphet had dropped precisions at very low and high concentrations (Fig. 3*C*). These results suggest that MsTargetPeaker is more robust against background noise and interference, especially under low-abundance conditions. This robustness may improve detection and quantification limits for low-concentration peptides.

To assess the consistency between automated and manual quantification, we next examined whether the area ratios from tool-selected peaks aligned well with manual annotations. Concordance was evaluated using PCC, SPC, and MAAPE. Figure 3*D* shows the trends across all 6300 peak groups, comparing each tool’s results with manual annotations. MsTargetPeaker achieved PCC, SPC, and MAAPE values of 0.9996, 0.9989, and 0.0110, respectively. For mProphet, these metrics were 0.9965, 0.9963, and 0.0098, whereas DeepMRM obtained 0.9977, 0.9978, and 0.0115. Overall, MsTargetPeaker showed the highest correlation with manual annotations (PCC and SPC) and a low MAAPE error, indicating strong agreement in peak area ratios.

Last, to further evaluate the quality of the selected peak regions, we compared TMSQE scores across manual annotations, mProphet, DeepMRM, and MsTargetPeaker. We used TMSQE as a quantitative and largely objective quality metric because it was established using an unsupervised learning approach with descriptive functions learned from our collected datasets of targeted proteomics, rather than arbitrary rules. As shown in Figure 3*E*, mProphet produced slightly lower TMSQE scores than manual annotations, whereas DeepMRM produced slightly higher scores. MsTargetPeaker also showed higher scores with lower variability.

### Performance Evaluation on External Datasets

**Fig. 4:**
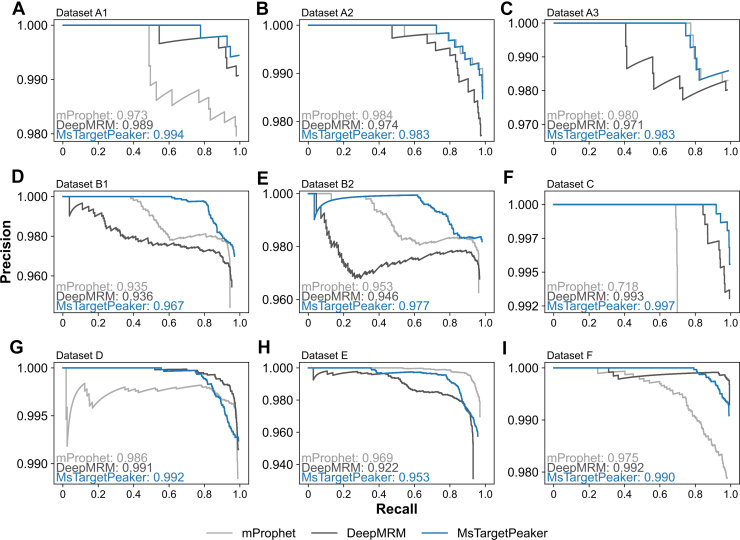
**Precision–recall curves across nine datasets.** The precision–recall curves illustrate the trade-off between precision and recall across varying threshold values and assess each tool’s ability to identify true target peak regions. Average precision (AP), calculated as the area under the precision–recall curve, summarizes overall performance. Higher AP values indicate better balance between precision and recall in detecting true peak regions. *A*–*I*, show the curves for mProphet (*light gray lines*), DeepMRM (*dark gray lines*), and MsTargetPeaker (*blue lines*), along with their respective AP values, for the nine datasets listed in Table 2, in the same order. *F*, the precision is capped at 0.992 to compare between MsTargetPeaker and DeepMRM, as mProphet has a performance degradation in this dataset.

We assessed the generalizability of MsTargetPeaker using nine external datasets derived from independent studies (20, 21, 22, 23, 24, 25), as listed in Table 2. Peak regions identified by mProphet, DeepMRM, and MsTargetPeaker were compared against published reference annotations. Using the same evaluation strategy as for the response curve data, we calculated AP for detection performance and assessed the concordance of peak area ratios using PCC, SPC, and MAAPE. The precision–recall curves for the nine datasets are shown in Figure 4, and these curves show that MsTargetPeaker consistently achieves higher precision at higher recall levels, leading to improved AP values across all datasets. In addition, the curves of MsTargetPeaker generally exhibit a smooth, monotonic decline, suggesting stable performance across thresholds and reflecting a well-designed reward function.

**Table 2:** Performance comparison among mProphet, DeepMRM, and MsTargetPeaker across nine datasets

Dataset	#Targets	#Peak group	mProphet				DeepMRM				MsTargetPeaker				Type	Reference
			AP	PCC	SPC	MAAPE	AP	PCC	SPC	MAAPE	AP	PCC	SPC	MAAPE		
A1	60	540	0.973	0.965	0.975	0.118	0.989	0.988	0.994	0.118	0.994	0.990	0.993	0.123	MRM	(20)
A2	57	786	0.984	0.991	0.991	0.054	0.974	0.961	0.976	0.102	0.983	0.976	0.981	0.092		
A3	59	354	0.980	0.985	0.981	0.037	0.971	0.956	0.956	0.089	0.983	0.985	0.972	0.076		
B1	63	2732	0.935	0.993	0.991	0.031	0.936	0.952	0.976	0.062	0.967	0.991	0.991	0.052		(21)
B2	67	2975	0.953	0.987	0.990	0.031	0.946	0.985	0.991	0.060	0.977	0.994	0.997	0.050		
C	23	1584	0.718	0.964	0.990	0.036	0.993	0.995	0.992	0.009	0.997	0.995	0.991	0.008		(22)
D	141	9952	0.986	0.989	0.982	0.042	0.991	0.990	0.988	0.033	0.991	0.993	0.990	0.026		(23)
E	74	4686	0.968	0.991	0.993	0.019	0.922	0.957	0.968	0.061	0.953	0.966	0.978	0.060	PRM	(24)
F	94	3814	0.975	0.998	0.998	0.009	0.992	0.997	0.997	0.018	0.992	0.997	0.997	0.022		(25)
Summary		Mean	0.941	0.985	0.988	0.042	0.968	0.976	0.982	0.061	0.982	0.987	0.988	0.057		
		SD	0.085	0.012	0.007	0.031	0.027	0.019	0.014	0.037	0.014	0.010	0.009	0.036		

These metrics (mProphet, DeepMRM, and MsTargetPeaker) include AP, PCC, SPC, and MAAPE.

AP, average precision; MAAPE, mean arctangent absolute percentage error; PCC, Pearson's correlation coefficient; SPC, Spearman’s rank correlation coefficient.

The resulting light-to-heavy area ratios of peaks identified by MsTargetPeaker showed high concordance with public references, achieving PCC and SPC values of 0.9875 ± 0.0103 and 0.9878 ± 0.0088, respectively. These results indicate better alignment with public references compared with mProphet and DeepMRM, as summarized in Table 2. Correlation plots for all nine datasets are presented in Supplemental Fig. S4.

**Fig. 5:**
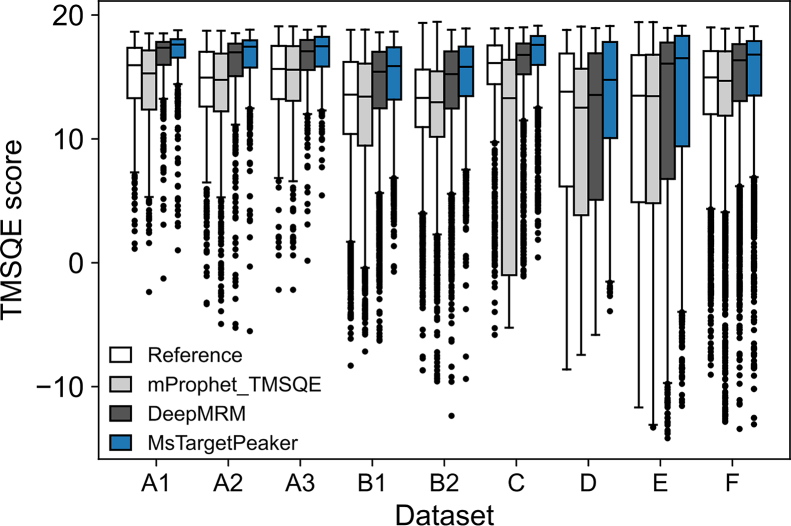
**Distribution of TMSQE quality scores across nine external datasets.** Boxplots show the distributions of TMSQE scores for peaks selected from public references (*white*), mProphet (*light gray*), DeepMRM (*dark gray*), and MsTargetPeaker (*blue*). Each dataset listed in Table 2 is represented by a group of three boxplots shown side by side for comparison. *Boxes* indicate interquartile ranges (IQRs), with median values shown as *horizontal lines*; *whiskers* extend to 1.5× IQR, and outliers beyond this range are shown as individual points.

We further evaluated peak quality using TMSQE scores (Fig. 5). DeepMRM identified peaks with higher scores than the public references, and MsTargetPeaker generally achieved higher scores and exhibited reduced variability. The distributions of quality scores across stratified intervals of area ratios further support the overall higher quality of peaks identified by MsTargetPeaker (Supplemental Fig. S5). This result is expected because MsTargetPeaker searches and optimizes reward scores that include a TMSQE quality component, which directly favors peaks with higher TMSQE quality. To examine this dependence, we performed an ablation in which the TMSQE component was removed during training and inference. In this ablation setting, MsTargetPeaker generally produced lower TMSQE scores than DeepMRM and showed decreases in AP, PCC, and SPC compared with the full model (Supplemental Figs. S6 and S7). Therefore, the TMSQE comparison reflects the intended effect of our reward design, and the ablation results further show that incorporating TMSQE also supports overall performance measured by AP, PCC, and SPC. Together, these results indicate that MsTargetPeaker improves peak identification precision, and that incorporating TMSQE into the reward function contributes to both higher TMSQE quality and better overall performance.

In addition to the TMSQE ablation, we performed further ablation analyses to assess the contribution of other design components in MsTargetPeaker. Removing individual reward components led to modest decreases in AP, PCC, and SPC (Supplemental Fig. S8). Among these components, peak compactness had the largest impact on AP because it influences peak width, which directly affects the IoU used in AP calculation. When the peak compactness term was removed, AP decreased by ∼10% on average across the nine datasets. In addition, increasing individual component weights by applying an exponent of two led to very small (<1%) relative decreases in AP, PCC, and SPC (Supplemental Fig. S9). Overall, these results indicate that MsTargetPeaker is generally robust to removing individual reward components or modestly varying component weights.

We further assessed how limiting the number of transition pairs in the observation matrix affected the final peak results during inference, as the observation matrix currently supports up to 20 transition pairs. When more than 20 transition pairs are available, we provide a random subset of transition pairs to the agent at each timestep so that different subsets take turns being included in the observation matrix. Under this setting, peak identification performance was not noticeably affected even when the observation matrix contained only two transition pairs at a time (Supplemental Fig. S10). This subsampling strategy can accommodate PRM experiments with many transitions and variable transition counts. Collectively, these results demonstrate that the proposed peak search approach effectively locates target signals, improves peak quality, and maintains generalizability across diverse test datasets.

### Cross-Sample Peak Boundary Consensus Enhances Precision

As transition ions from the same target peptide tend to elute within similar retention time windows across samples, cross-sample consensus plays a critical role in guiding the search toward correct peak regions, particularly when target signals are weak or ambiguous. During the search process, MsTargetPeaker incorporates boundary consensus profiles derived from peaks across samples of the same target peptide. These profiles are constructed from qualified peaks identified in earlier search rounds. By leveraging the multiround search strategy, MsTargetPeaker gradually accumulates reliable boundary information and refines peak regions over successive rounds.

**Fig. 6:**
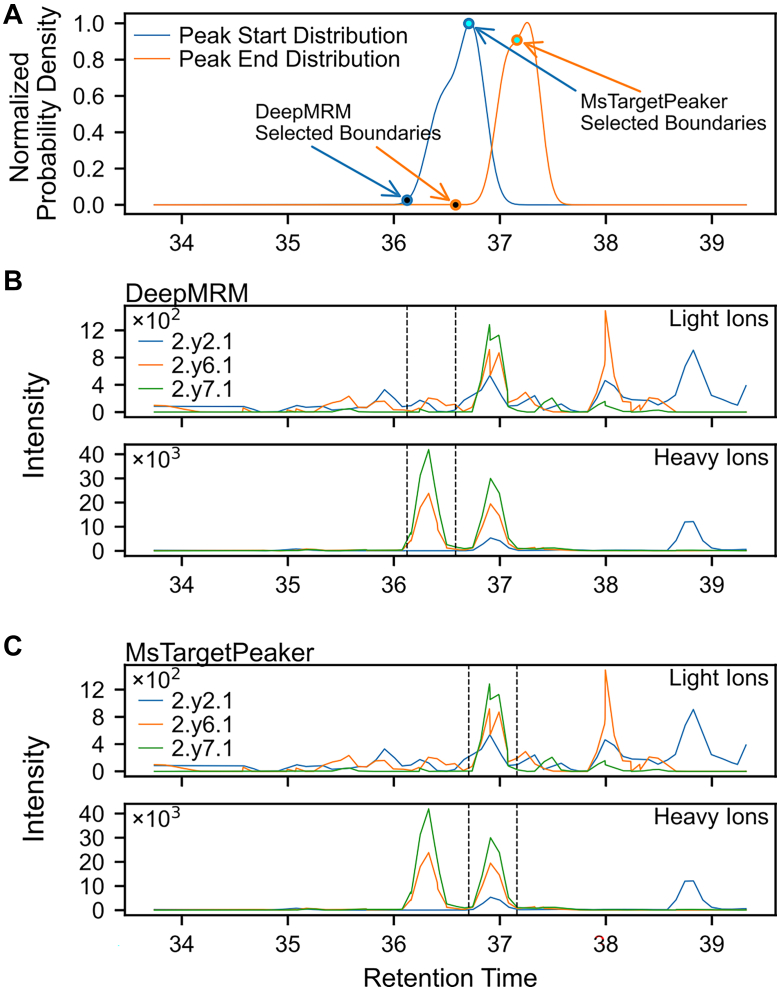
**Boundary consensus profile guides peak selection under ambiguous signal conditions.** The target peptide YMEDSTY[+80]YK is shown as an example to illustrate how cross-sample boundary consensus helps identify the correct peak region. *A*, a peak boundary consensus profile was constructed during peak identification. The boundary consensus profile is computed from confident peaks as smoothed retention-time density curves for peak start (*blue*) and peak end (*orange*), and it provides a soft prior in later MCTS rounds. Higher density values indicate more likely boundary locations for the target peak, as more confident peaks occur at nearby retention times. MsTargetPeaker uses this consensus profile to guide boundary selection toward positions that are consistent across samples. *B* and *C*, example of peak boundary selections by DeepMRM (*B*) and MsTargetPeaker (*C*). In this peak group, the reference heavy chromatograms contain two distinct peaks, and the *right peak* corresponds to the annotated target signal. The *left peak* is more intense and may therefore appear more prominent, which could contribute to its selection in *B*. In contrast, the consensus profile in *A* guides the later-round search toward boundary locations that are consistent across samples, thereby favoring the *right peak*, as shown in *C*. The boundaries selected in *B* and *C* are overlaid in *A* as circular markers (*black* for DeepMRM and *cyan* for MsTargetPeaker). *Arrows* highlight the corresponding start and end boundaries selected by each tool. MCTS, Monte Carlo tree search.

To illustrate the benefits of this approach, we selected an example and compared the peak boundaries identified by DeepMRM and MsTargetPeaker (Fig. 6). MsTargetPeaker leverages the cross-sample peak boundary consensus profile using the probability density function to establish empirical retention-time distributions of peak start and peak end from the identified high-scoring peaks of each target. The consensus profile can be incorporated into the reward function to guide the search toward regions more likely to contain true target signals. When endogenous light signals are absent and reference heavy signals are obscured by interference, the boundary consensus provides crucial guidance for locating target peaks. As a result, the consensus mechanism helps ensure that the same correct peak region is consistently identified across all samples of the peptide, thereby enabling robust and precise identification even under low-abundance and high-interference conditions.

### MsTargetPeaker Workflow Generates Peak Results With Interpretable Diagnostic Reports

**Fig. 7:**
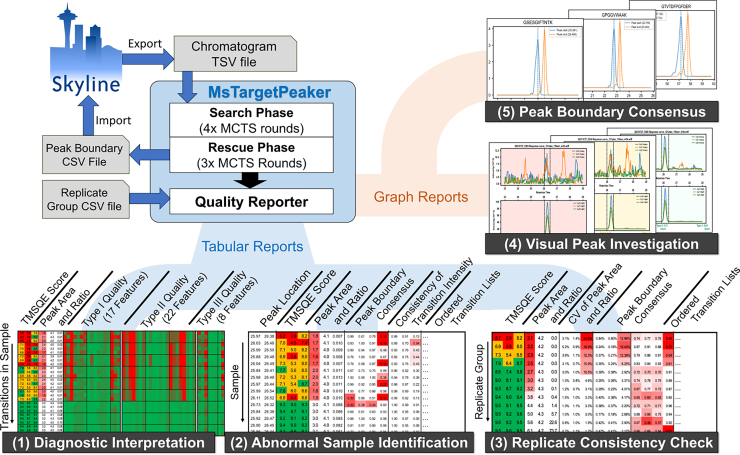
**Usage workflow of MsTargetPeaker and diagnostic reports.** MsTargetPeaker takes chromatogram data in the file of the tabular-separated values (TSVs) format that can be exported *via* Skyline. After the target peak search, the output peak boundary file can be imported into Skyline to update peak boundaries. In addition, MsTargetPeaker comes with a quality reporter that can generate interpretable peak reports for efficient quality assurance. The five types of reports can assist users to assess (1) individual transition quality, (2) overall peak group quality, (3) replicate consistency quality, (4) visual examinations of peak groups, and (5) the distributions of peak boundary from cross-sample peak groups for each target peptide.

To enhance the quality assurance of peak identification, MsTargetPeaker generates diagnostic reports that provide interpretable quality metrics for each identified peak region. Peak identification may fail when chromatograms lack qualified peak regions or contain strong interference signals that resemble desirable peak characteristics, potentially misleading the search process. These challenges are particularly common in low-abundance targets and complex biological samples. The diagnostic reports assist users in detecting low-quality or problematic cases, helping to mitigate such issues and improve result reliability. This quality control process can be incorporated into routine peak identification workflows with Skyline (Fig. 7).

The diagnostic reports consist of five outputs: (1) transition-level quality reports that list TMSQE scores and underlying quality features for each monitored transition; (2) peak group-level reports that highlight low-confidence peak groups based on aggregated transition-level metrics; (3) replicate consistency reports that assess variation across replicate samples; (4) chromatogram visualizations that display identified peak regions with color-coded backgrounds (red, yellow, and green) indicating poor, acceptable, and high-quality peaks; and (5) boundary consensus profiles that illustrate normalized probability density functions of start and end retention times across samples for each target peptide. The first three outputs are provided in Excel spreadsheet format to support filtering and inspection using color-coded metrics, whereas the latter two are provided as PDF visualizations to support visual evaluation of peak quality.

We highlight five key elements of these diagnostic reports that facilitate the identification of cases potentially requiring manual re-evaluation. First, TMSQE scores enable quantitative assessment of peak quality at the levels of individual transitions, peak groups, and replicate samples, providing a practical basis for filtering out low-confidence results. Second, cross-sample peak boundary consensus helps detect peak regions with inconsistent or atypical boundary locations, with higher consensus values indicating better agreement across samples for each target peptide. Third, the consistency of light-to-heavy area ratios is evaluated using multiple metrics, including the dot product, normalized spectral contrast angle (26), and PairRatioConsistency. Fourth, fragment ion rankings based on peak intensity, quality scores, and consistency metrics assist in filtering out abnormal transitions and selecting suitable quantifier ions. Finally, when subsets of target peptides are eluted at shifted retention times, a multimodal distribution will be established to address the batch effects and can be visualized in our diagnostic report (Fig. 7). MsTargetPeaker's diagnostic reports offer a comprehensive and effective solution for chromatographic quality assurance.

### Execution Time and Acceleration of MsTargetPeaker

The running time of MsTargetPeaker depends on peak quality. For chromatograms with clear target signals and minimal interference, the search typically completes within a single round and takes less than 1 min per peak group. In contrast, ambiguous or low-quality peaks may require all seven rounds to identify the correct peak regions. Detailed running times for different numbers of search rounds are provided in Supplemental Table S3. To accelerate the search, we tested reducing the number of MCTS cycles. As shown in Table 3, when the number of cycles was reduced by 90%, the average AP value dropped by only 0.07 ± 0.18% across the nine testing datasets, suggesting that substantial speed gains can be achieved with minimal performance loss (see detailed performance reduction in Supplemental Table S4). Given the small AP change observed after substantially reducing the cycle count, we expect that increasing the number of cycles beyond the full-cycle setting will provide little performance gain, while the running time increases with the number of cycles. Although the full-cycle setting was used in our main analyses (Table 1), the 90%-reduced setting is used as the default in MsTargetPeaker to improve efficiency, allowing each peak group to be processed within 20 s per search round. Users can increase the number of cycles *via* the configuration file to obtain more robust and fine-grained results. MsTargetPeaker also supports parallelization. On a single machine, it can accelerate the search by utilizing multiple CPU cores. For multimachine environments, it provides built-in tools to split input chromatogram files into smaller segments and to combine peak results after distributed execution.

**Table 3:** Performance reduction for fewer MCTS cycles

MCTS cycle number reduction (%)	AP change	PCC change	SPC change	MAAPE change
50	−0.05 ± 0.09%	0.06 ± 0.24%	0.01 ± 0.05%	−0.59 ± 1.65%
80	−0.05 ± 0.08%	0.07 ± 0.21%	−0.02 ± 0.06%	−0.69 ± 2.17%
90	−0.07 ± 0.18%	0.05 ± 0.24%	−0.04 ± 0.09%	0.17 ± 1.14%
95	−0.12 ± 0.16%	−0.12 ± 0.57%	−0.08 ± 0.32%	−0.15 ± 3.24%

AP, average precision; MAAPE, mean arctangent absolute percentage error; MCTS, Monte Carlo tree search; PCC, Pearson's correlation coefficient; SPC, Spearman’s rank correlation coefficient.

## Discussion

In this study, we present MsTargetPeaker, a quality-aware peak search procedure that combines a deep reinforcement learning agent with MCTS to locate target peak regions for subsequent area integration and quantification. This approach employs a reward function that integrates TMSQE quality scores with additional components to capture desirable peak characteristics, guiding the search toward high-quality peak regions. We demonstrated the high AP of MsTargetPeaker across a wide range of concentrations and its strong generalizability across diverse datasets. By incorporating cross-sample boundary consensus into the search, we showed that this strategy maintains robust peak identification in the presence of ambiguous signals. After peak identification, MsTargetPeaker generates interpretable quality reports that help users identify cases that may require manual review. We envision that MsTargetPeaker can substantially advance targeted proteomics by offering a more reliable and automated solution for peak identification with built-in quality control.

A main feature of MsTargetPeaker is the transparency of its reward function calculation. The search parameters defined in the configuration file can be adjusted based on user preferences. By reshaping the reward function with different parameter sets, the search can be fine-tuned toward desired peak characteristics. For example, applying stricter criteria by increasing the weight of PairRatioConsistency can exclude peaks with inconsistent transition ratios. However, overly stringent criteria may lead to selecting only ideal peaks and overlooking valid but imperfect signals. Low-abundance targets often produce peaks with some degree of transition ratio inconsistency, asymmetry, or interference, making them more likely to be excluded under strict configurations. In this study, we used empirically derived parameters as the default setting in MsTargetPeaker, favoring high-quality peaks while tolerating imperfections. With this established search standard and the accompanying quality reports, we may expand detection limits and improve the quantifiable range of target peptides.

Currently, MsTargetPeaker is limited to peak groups with paired light and heavy ion signals, such as spike-in stable isotope–labeled references. The current implementation relies on our reward function to define desirable peak characteristics. Under this reward design, a peak group receives a high reward score when the selected region contains a compact peak with good integrity, is supported by strong reference signals, and achieves a high TMSQE score. These four reward components account for most of the peak inference performance (Supplemental Fig. S8). In addition, individually increasing the weight of each of the four components slightly reduced performance across the nine testing datasets (Supplemental Fig. S9), suggesting that the reward design is reasonably balanced. Therefore, MsTargetPeaker can, in principle, be applied to any peak group that can be evaluated under the same quality criteria.

During peak inference, MsTargetPeaker may be extended by adding new scoring components to the reward function, such as signal-to-noise ratio or ion mobility–derived features, without retraining the agent. This flexibility is supported by our ablation results: when TMSQE is removed from both agent training and peak inference, performance decreases, but the loss is recovered when TMSQE scoring is reintroduced only at inference (Supplemental Fig. S7). However, this design also introduces a trade-off. Because TMSQE is a quality-oriented metric, optimizing a reward that includes TMSQE can favor peak regions that score well under this definition of quality. The relatively low TMSQE scores for the manual, mProphet, and DeepMRM results likely reflect those outputs were not optimized for TMSQE. In contrast, at higher area ratios, the scores tend to be saturated because high-concentration conditions usually produce distinct and high-quality peaks (Supplemental Fig. S5). This design aligns the search objective with peak quality, but it also means that the inferred peaks and downstream quantification decisions are influenced by the chosen quality scheme. Therefore, for new assay types or workflows with different peak-quality assumptions, the reward components and their weights may require adjustment or extension.

A second trade-off arises from the cross-sample boundary consensus. This term is built from empirical boundary distributions (peak start and peak end) estimated for each peptide across the analyzed samples. It can represent batch effects as multimodal boundary patterns. In our evaluation, removing this component did not noticeably change overall performance (Supplemental Fig. S8), likely because ambiguous cases were rare in the tested datasets. Nevertheless, the consensus term serves as a safeguard for ambiguous cases and reduces occasional inconsistent peak picking across samples, as shown in Figure 6. The trade-off is that the consensus profile is estimated from the analyzed sample set. When the sample size is small, when few confident peak groups are available to estimate the consensus, or when the sample set spans many experimental conditions or batches, the estimated consensus density may be less reliable. In such cases, MsTargetPeaker can flag inconsistent peak boundaries through low consensus probability density in the diagnostic reports for further review.

To facilitate full automation of peak identification and target quantification in targeted proteomics, MsTargetPeaker offers precise peak search and generates quality reports. However, the current version does not implement a full quantification workflow. As a practical starting point, we recommend exporting chromatograms from Skyline as TSV files, running MsTargetPeaker with the default settings, using the diagnostic reports to identify problematic cases, and importing the generated peak-boundary CSV file back into Skyline for downstream analysis (Fig. 7). Based on the diagnostic reports, low-scoring peak groups, highlighted in red or yellow, can be excluded from quantification or flagged for follow-up review. Although criteria for handling such cases are not yet well defined, these reports provide a foundation for developing downstream procedures to handle low-scoring peaks and reduce manual intervention. Future work will focus on integrating peak-level quantification calculations and decision approaches for handling low-scoring peaks to further streamline quantification workflows. This approach can support the establishment of a consistent and fully automated workflow for precise target quantification. Such capability has strong potential for clinical applications, where high throughput and standardized quantification are critical for biomarker validation and routine diagnostics.

## Data Availability

MsTargetPeaker can be installed from the Python Package Index. Its source code and user manual are publicly available on GitHub at https://github.com/chiyang/MsTargetPeaker. The response curve dataset is available on Panorama Public with the access link (https://panoramaweb.org/dss105rrc.url).

## Supplemental Data

This article contains supplemental data (6, 8, 15, 20, 21, 22, 23, 24, 25).

## Conflict of Interest

The authors declare no competing interests.
