# Supplementary material for: MsTargetPeaker: A Quality-Aware Deep Reinforcement Learning Approach for Peak Identification in Targeted Proteomics
Source: Mol Cell Proteomics. 2026 Feb 2;25(3):101523. doi: 10.1016/j.mcpro.2026.101523 (PMC12966724; doi:10.1016/j.mcpro.2026.101523)
Supplement: Supplemental Figures [file mmc1.docx]

**Supplemental Information**

**MsTargetPeaker: a quality-aware deep reinforcement learning approach for peak identification in targeted proteomics.**

Chi Yang^1*^, Yung-Chin Hsiao^1,2,3^, Chi-Ching Lee^4,5^, Lichieh Julie Chu^1,2,3^, Ta-Sen Yeh^3,6^, Ping-Chang Cheng^1^, Petrus Tang^1,7,8^, and Jau-Song Yu^1,2,9,10*^

**Affiliations:**

^1^Molecular Medicine Research Center, Chang Gung University, Taoyuan, Taiwan

^2^Graduate Institute of Biomedical Sciences, College of Medicine, Chang Gung University, Taoyuan, Taiwan

^3^Department of Surgery, Chang Gung Memorial Hospital, Linkou, Taiwan.

^4^Department of Computer Science and Information Engineering, Chang Gung University, Taoyuan, Taiwan

^5^Genomic Medicine Core Laboratory, Chang Gung Memorial Hospital, Linkou, Taiwan

^6^College of Medicine, Chang Gung University, Taoyuan, Taiwan.

^7^Department of Parasitology, College of Medicine, Chang Gung University, Taoyuan, Taiwan

^8^Molecular Infectious Disease Research Center, Chang Gung Memorial Hospital, Linkou, Taiwan

^9^Department of Otolaryngology-Head and Neck Surgery, Chang Gung Memorial Hospital, Taoyuan 33305, Taiwan.

^10^Research Center for Food and Cosmetic Safety, College of Human Ecology, Chang Gung University of Science and Technology, Taoyuan, Taiwan

*****Corresponding Authors: chiyang@mail.cgu.edu.tw and yusong@mail.cgu.edu.tw

**Table of Content**

[Figure S1. Illustration of the peak-group state and observation matrix before and after peak-boundary movement. 3](#_Toc218116326)

[Figure S2. The calculation derived for peak integrity. 4](#_Toc218116327)

[Figure S3. Score transformation functions used in the reward function. 5](#_Toc218116328)

[Figure S4. Peak area ratio correlations with manual annotations across the nine external datasets. 6](#_Toc218116329)

[Figure S5. TMSQE quality score distributions in the nine external datasets. 7](#_Toc218116330)

[Figure S6. TMSQE score distributions across the nine testing datasets with the TMSQE component removed from the reward function. 8](#_Toc218116331)

[Figure S7. Restoring TMSQE during peak inference recovers performance when TMSQE is excluded from training. 9](#_Toc218116332)

[Figure S8. Performance impact after removing individual reward components. 10](#_Toc218116333)

[Figure S9. Performance impact when varying individual components. 11](#_Toc218116334)

[Figure S10. Slight performance changes when limiting the number of transition signals in the observation matrix. 12](#_Toc218116335)


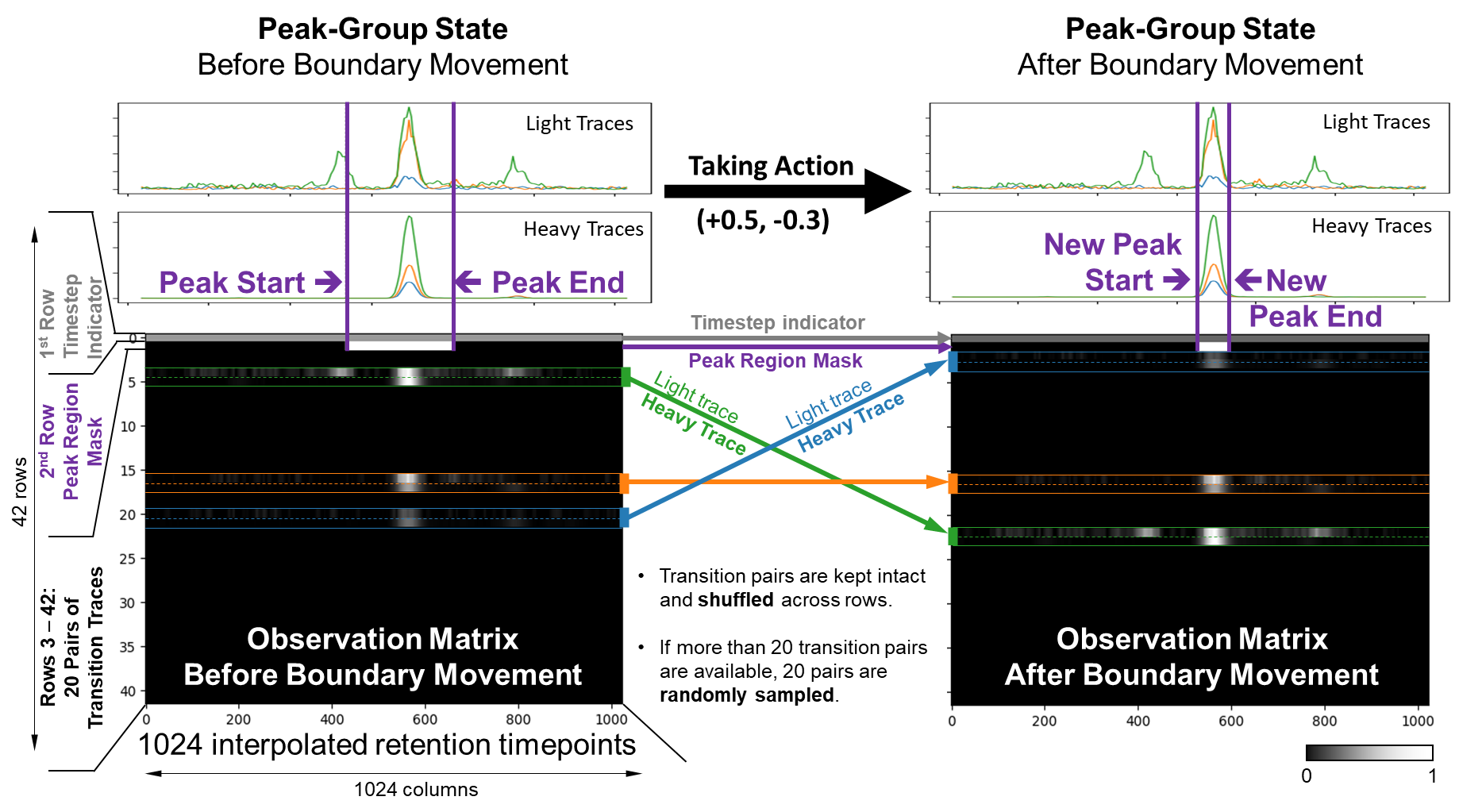


# Figure S1. Illustration of the peak-group state and observation matrix before and after peak-boundary movement.

Each observation matrix is a 42 x 1024 matrix and is a numerical representation of a peak-group state. The 1024 columns are evenly spaced retention-time bins obtained by linear interpolation of the chromatogram. The 42 rows consist of two control channels and 40 signal channels. Row 1 is a timestep indicator that repeats a single value across all columns to encode the remaining number of moves, and row 2 is a peak-region mask with 1 inside the current peak region and 0 outside. Rows 3–42 store intensity traces for up to 20 light/heavy transition pairs, with two adjacent rows per pair. When fewer than 20 pairs are available, the unused rows are set to zero. When more than 20 pairs are available, only 20 pairs are shown at each timestep. These 20 pairs are randomly resampled every time the observation matrix is rebuilt. We highlight this design because it gradually exposes different subsets of transition pairs to the agent across timesteps rather than always showing a fixed subset. At each timestep, the environment rebuilds the observation matrix to reflect updated peak boundaries, and the order of the transition-pair rows is randomly shuffled so that specific pairs are not tied to fixed row indices. This representation allows the environment to handle varying numbers of transition pairs across target peptides.

Before being passed to the agent, all intensity values in the observation matrix are preprocessed in four steps. First, raw intensities below 1 are set to 1 to allow valid square-root calculations. Second, a square-root transformation is applied. Third, for each transition ion, the transformed trace is min–max normalized to the range [0, 1]. Finally, to suppress very low-level noise, we computed the 30th percentile of normalized intensity values within the observation matrix. Values below this threshold were set to zero. The square-root transformation makes low-intensity peak regions more distinguishable to the agent, and zeroing very small values prevents the agent from focusing on noisy baseline regions, which improves learning efficiency.


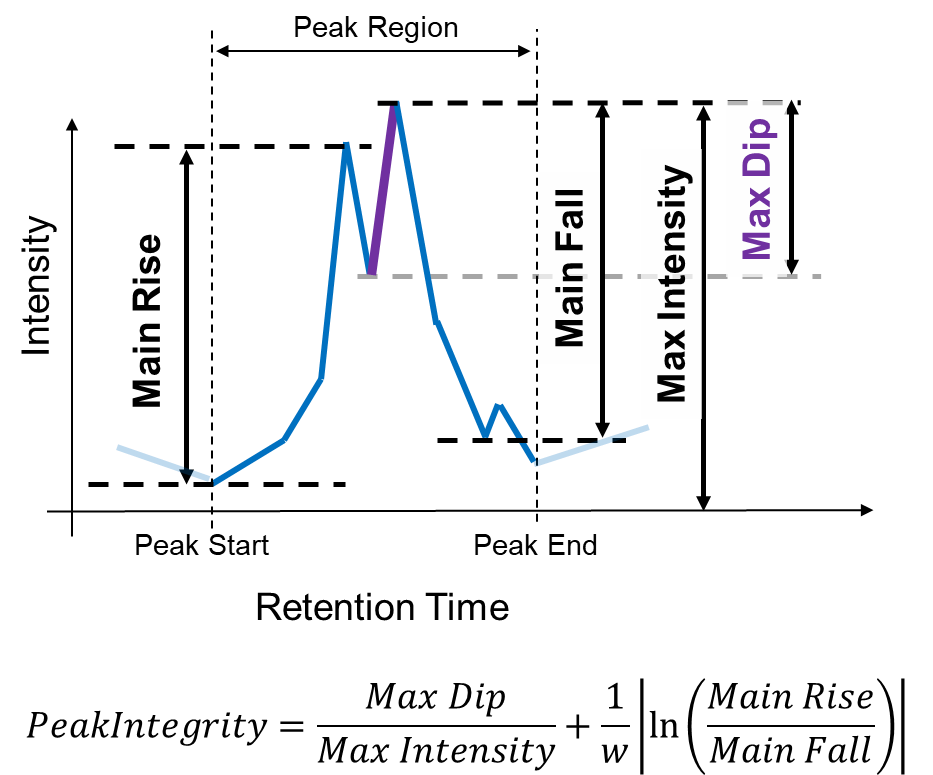


# Figure S2. The calculation derived for peak integrity.

The original modality metric calculation in TargetedMSQC only concerns about the max dip of the peak as shown in the first term of our version of the peak integrity formula. We additionally consider the main rise and main fall of the peak to address the integrity of an ideal peak shape. A perfect peak has no dip and has equal main rise and fall. The peak integrity for a good peak should be thus close to zero. Since we may not maintain the integrity of the desirable peak shape after excluding interference signals within the peak region, the main rise and fall are not always required to be similar or equal. We reduced the weight for such rise-fall equality. In this study, we set the weight w to three to have one-third contribution to the final peak integrity calculation. Consequently, the resulting identified peak can maintain its shape integrity, while allowing the integrity to be compromised when necessary to exclude interference signals from the peak region.

**
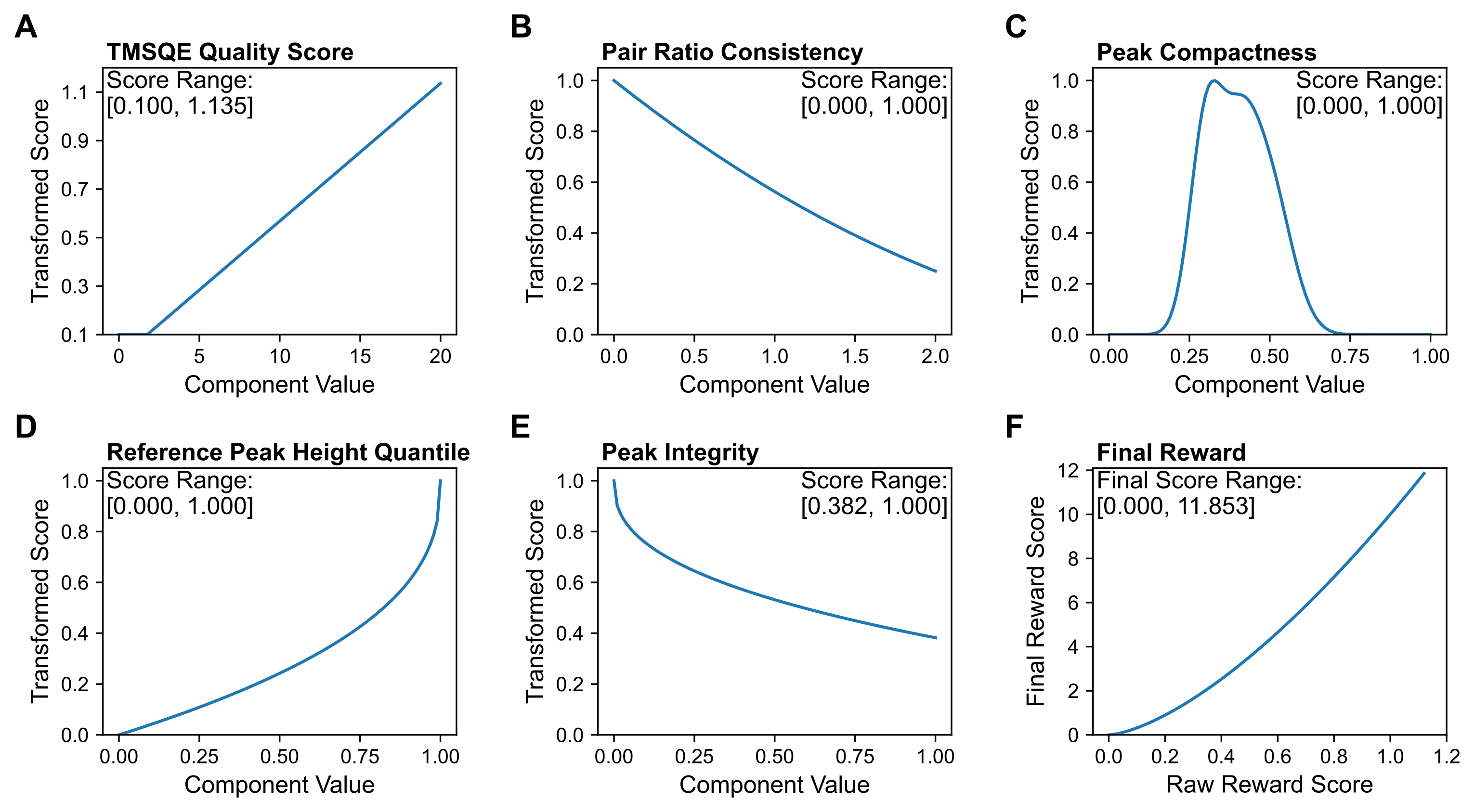
**

# Figure S3. Score transformation functions used in the reward function.

This figure shows the transformation functions applied to five reward components: (A) TMSQE quality score, (B) PairRatioConsistency, (C) PeakCompactness, (D) ReferencePeakHeightQuantile, and (E) PeakIntegrity. Each component has its own value range and direction that reflect desired peak characteristics. The transformation functions shown in this figure convert these values into unified, directed scores, where higher values indicate more favorable peak regions. The raw reward score is calculated as the product of these transformed scores, together with two additional components that do not require transformation: a binary indicator of signal existence and a peak boundary consensus score, as both already range from 0 to 1. The raw reward score is then transformed into the final reward score by scaling it by 10 and applying a power transform with an exponent of 1.5 (F). This transformation provides a mild gradient that encourages the reinforcement learning agent to select peaks with higher scores. For detailed calculations, please refer to our source code available on GitHub (<https://github.com/chiyang/MsTargetPeaker>).

**

**

# Figure S4. Peak area ratio correlations with manual annotations across the nine external datasets.

We compared the light-to-heavy area ratios of chromatographic peaks among tool selections and public reference. The x axis for each of these nine plots represent the log2-transformed area ratios from the reference, while the y axis represents the ratios from accepted peak groups of mProphet, DeepMRM, and MsTargetPeaker results. Next to each tool name, we report the number of accepted peak groups that passed the score cutoff that maximized the F1 score. The diagonal line depicts the line where x values equal to y, and points near this diagonal line indicates high alignment with the reference. The nine plots from A to I were presented in the same order with the nine external datasets as listed in **Table S1** and **Table 2** in the main text. The number of accepted peak groups for each tool is shown in each panel. For each tool, accepted peaks were defined using a tool-specific score cutoff selected to maximize that tool’s F1 score in the precision–recall analysis. Please refer to **Table2** in the main text for the detailed correlation values and the mean arctangent absolute percentage errors (MAAPE).

**
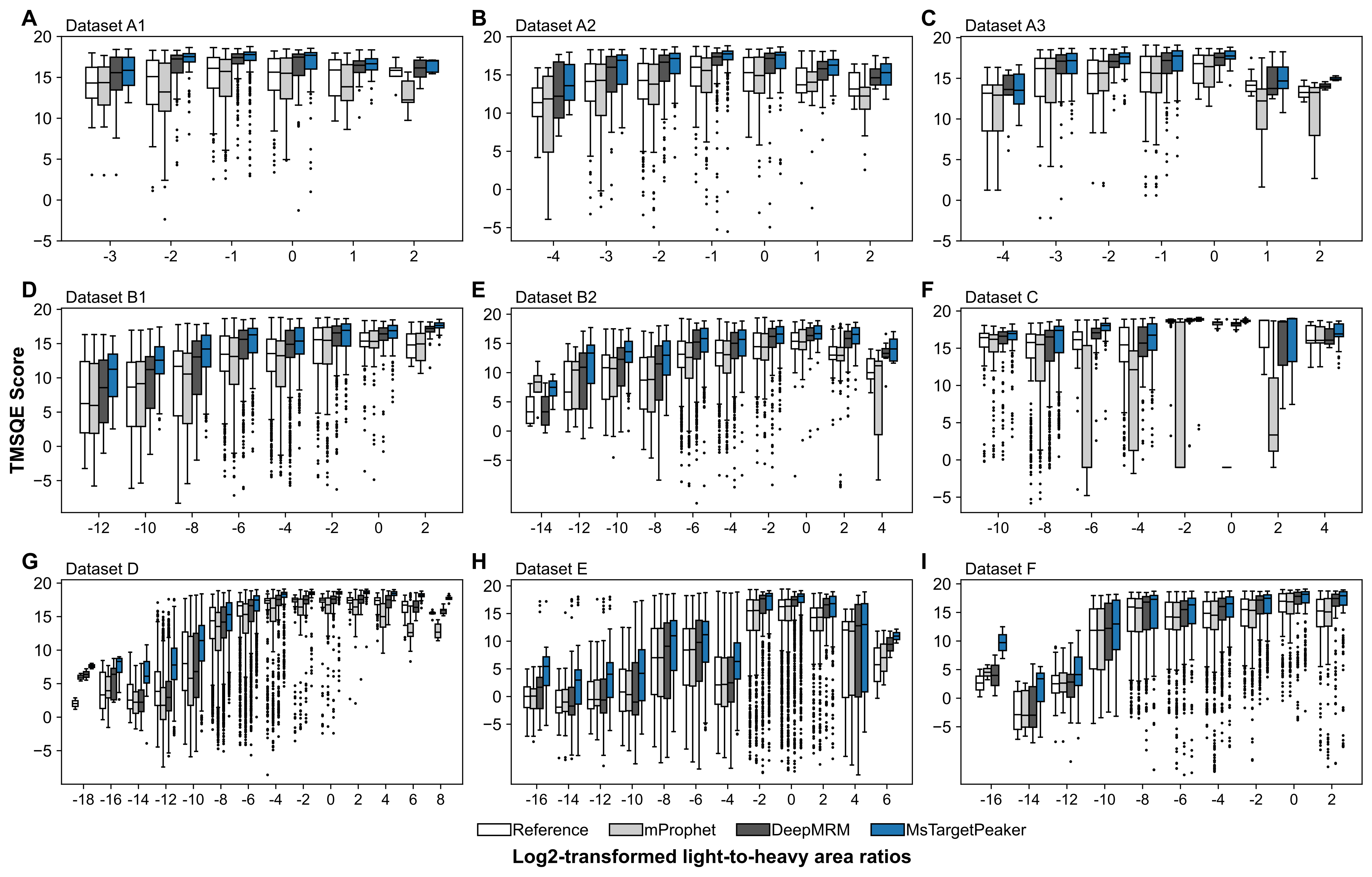
**

# Figure S5. TMSQE quality score distributions in the nine external datasets.

To investigate the peak quality score distributions over various amount of the light-to-heavy area ratios, we compared the quality scores from the selected peaks of mProphet, DeepMRM, and MsTargetPeaker with the scores from published references. Similarly, the nine boxplots present the score distributions in the same order with the nine external datasets as listed in **Table S1** and **Table 2** in the main text. Each lane in the boxplot shows the distribution of TMSQE quality scores within an interval of log₂-transformed area ratios. Each interval is defined by two adjacent x-axis labels: the current label indicates the lower bound, and the next label to the right indicates the upper bound. Panels


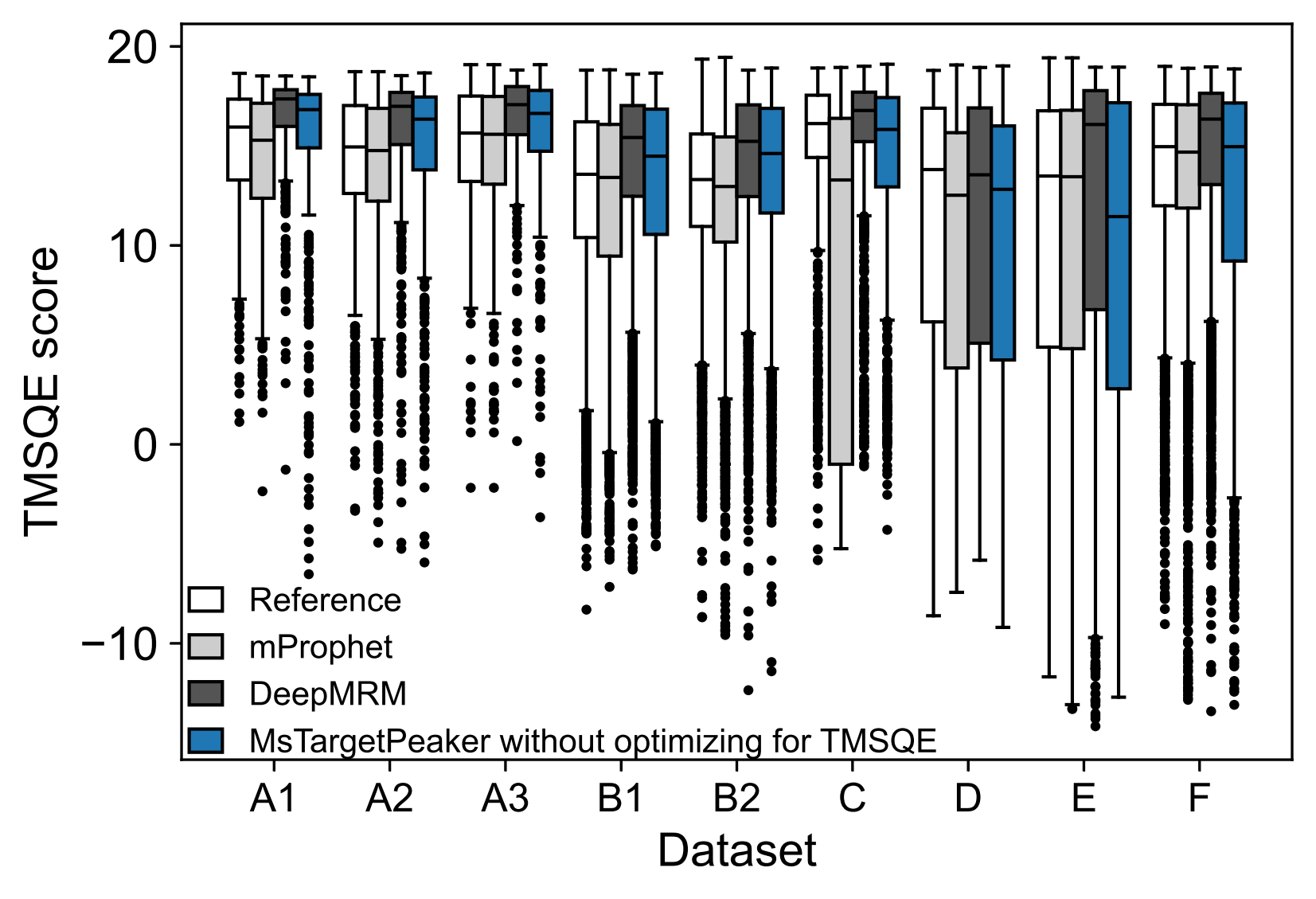


# Figure S6. TMSQE score distributions across the nine testing datasets with the TMSQE component removed from the reward function.

To assess the impact on the TMSQE scores without optimizing for TMSQE, we retrained the agent using a TMSQE-free reward function and applied the same peak identification workflow to the nine testing datasets. The figure shows the resulting TMSQE score distributions for four methods: reference peaks, mProphet, DeepMRM, and MsTargetPeaker. DeepMRM showed the highest overall TMSQE scores across the datasets, indicating that it can identify peaks with high TMSQE quality even though it was not designed to optimize TMSQE. In contrast, under the TMSQE-removed setting, MsTargetPeaker yielded lower TMSQE scores than DeepMRM, highlighting the importance of including the TMSQE component during reward optimization in MsTargetPeaker. This effect was most pronounced for dataset E, where MsTargetPeaker had the lowest median TMSQE score among the four methods.





# Figure S7. Restoring TMSQE during peak inference recovers performance when TMSQE is excluded from training.

Bars show the relative decrease (%) in average precision (AP), Pearson correlation coefficient (PCC), and Spearman’s rank correlation coefficient (SPC), compared with the default setting that uses TMSQE in both agent training and MCTS-based peak inference. In the left group, TMSQE was excluded during both agent training and peak inference, resulting in decreases in AP, PCC, and SPC. In the right group, TMSQE was excluded during agent training but restored in the reward function during peak inference, resulting in performance recovery. Error bars indicate variability across the nine testing datasets (mean ± SD).





# Figure S8. Performance impact after removing individual reward components.

We evaluated the contribution of each reward component by removing one component at a time and measuring the relative decrease (%) in average precision (AP), Pearson correlation coefficient (PCC), and Spearman’s rank correlation coefficient (SPC) relative to the full setting that includes all reward components across the nine testing datasets. Four components contributed to peak identification performance, as indicated by performance decreases when each was removed individually: TMSQE, peak compactness, peak integrity, and reference integrity. For most components, the performance decrease was small (<2.8%). The largest change was observed when peak compactness was removed, which reduced AP by ~10%, consistent with its influence on peak width and the IoU used in AP calculation. In contrast, removing the remaining three components had little effect on AP, PCC, or SPC. The signal existence component was designed to improve training efficiency by helping the agent skip low-signal regions. The minimal performance change after removing this component suggests that it may contribute less to peak identification during inference. Similarly, transition ratio consistency was introduced to guide training by helping the agent focus on co-eluted transition pairs with similar light–heavy ratio ordering. This information may be partly captured by TMSQE, which may explain its limited impact when removed. Lastly, cross-sample boundary consistency may contribute little because ambiguous peaks are rare in these datasets. Nevertheless, we retain this component as a safeguard for difficult cases.





# Figure S9. Performance impact when varying individual components.

To assess sensitivity to component weighting, we increased the weight of each of the four components highlighted in **Figure S8** (TMSQE, peak integrity, reference peak height quantile, and peak compactness) one at a time by applying an exponent of 2 to that component in the multiplicative reward function while keeping all other components unchanged. After up-weighting, all four modifications led to small decreases in AP, PCC, and SPC compared with the unmodified reward function. This pattern suggests that the default reward components are reasonably balanced, and that further emphasizing any single component can slightly disrupt the overall objective. Bars show the relative decrease in AP, PCC, and SPC. Error bars indicate the standard deviation across the nine datasets.


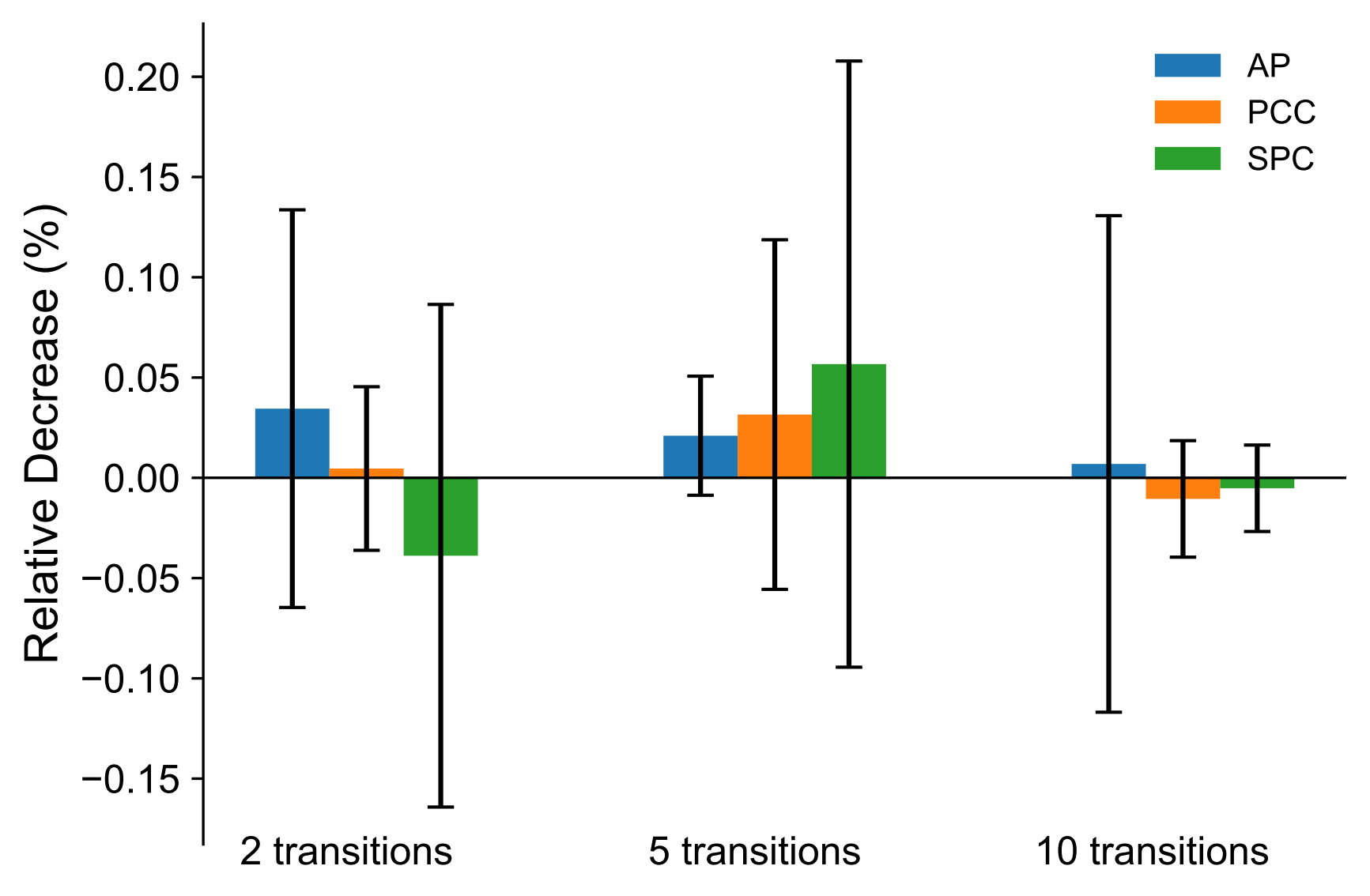


# Figure S10. Slight performance changes when limiting the number of transition signals in the observation matrix.

The default observation matrix can include up to 20 transition pairs per timestep. Here, we limited the number of transition pairs to 2, 5, or 10 by subsampling from the available transitions, while keeping all other settings unchanged. The 2-transition setting was evaluated across all nine datasets, and the 5- and 10-transition settings were evaluated only on the two PRM datasets (datasets E and F) because only these datasets contained more than five transition pairs. Bars show the average relative decrease (%) in AP, PCC, and SPC compared with the default setting. Error bars indicate the standard deviation across the evaluated datasets.
